# Alleged Cause of Death from Nitrous Oxyd

**Published:** 1871-05

**Authors:** 


### ALLEGED CAUSE OF DEATH FROM NITROUS OXYD.

Dr. A. F. E., of Columbus, O., furnishes us the following statement in regard to the death of one of his patients, which it was alleged died from the effects of nitrous oxyd, administered as an anæsthetic:

"On January 10th, last, at 5 o'clock, P. M., Mr. Davis called at my office to have the ulcerated remains of an offending tooth extracted, and insisted upon having nitrous oxyd administered. Not being able to detect anything that indicated danger, I proceeded at once to administer the gas. He lacked the courage to inhale it. After several unsuccessful attempts, the gas was abandoned, and the roots extracted, Mr. D. being conscious all the time. He had inhaled of the gas not to exceed 1½ gallons, and after the operation, said he had felt no effects from the gas whatever.

Mr. D. had a subsequent illness of several days, but recovered sufficiently to be about and attend to business. A few days prior to his death, he went to Cincinnati on business and returned to Columbus, and had made his arrangements to start for Chicago, (his place of business,) the morning of his death, which took place February 2nd. He was sick but a few minutes. The deceased, I understand, was carrying at the time of his death, $30,000 life insurance, the greater part of which was taken out within the last few months, the last policy within a few weeks of his death. That would necessarily have submitted him to a pretty thorough examination by the examining physicians of the several companies in which he insured. They failed to detect the difficulty and accepted him.

The attending physician's affidavit reads thus: Died of heart disease, produced by inhaling laughing gas, etc.

Now, laughing gas is not an agent that will produce disorganization. Therefore, there must certainly have been some abnormal structure of that organ. Nitrous oxyd, like all other anæsthetics, is dangerous, and must be administered with discrimination. But it does not follow, that because anæsthetics are dangerous, that they must necessarily prove hurtful in all cases.

Considering the examination Mr. D. had passed by members of the medical profession, this particular case like others, would require superhuman skill to detect whether the subject might or might not have some organic defect or disease.

Now one of two things are true, Mr. D. must have disease of the heart, or the gallon and a half of gas killed him.

The gas was fresh, being made that day, and I have every reason to believe it was pure, for it was administered to other patients, with the most satisfactory results, as well as having taken it myself.

Strange to say, if a person has ever taken an anæsthetic, particularly laughing gas and dies subsequently, no matter how long after, the gas killed them or exerted a powerful influence in that direction. The simple fact that a person has taken an anæsthetic, does not immortalize them. Their chances of mortality, sooner or later, are just as good as they ever were. Some may say better. We will not presume to say.

